# Building competency to deal with environmental health challenges: experiences and a proposal

**DOI:** 10.3389/fpubh.2024.1373530

**Published:** 2024-11-18

**Authors:** Giovanni S. Leonardi, Ariana Zeka, Matthew Ashworth, Catherine Bouland, Helen Crabbe, Raquel Duarte-Davidson, Ruth A. Etzel, Nia Giuashvili, Özden Gökdemir, Wojciech Hanke, Peter van den Hazel, Paul Jagals, Ejaz Ahmad Khan, Piedad Martin-Olmedo, Joseph Pett, Ekaterine Ruadze, Maria Grazia Santamaria, Jan C. Semenza, Cecilia Sorensen, Sotiris Vardoulakis, Fuyuen Yip, Paolo Lauriola

**Affiliations:** ^1^UKHSA, London, United Kingdom; ^2^LSHTM, London, United Kingdom; ^3^University College London, London, United Kingdom; ^4^Institute of Environmental Science and Research Limited (ESR), Upper Hutt, New Zealand; ^5^The Child Health Research Centre, The University of Queensland, Brisbane, QLD, Australia; ^6^Université Libre de Bruxelles (ULB)—Ecole de Santé Publique, Brussels, Belgium; ^7^Milken Institute School of Public Health, The George Washington University, Washington, DC, United States; ^8^National Center for Disease Control and Public Health, Tblisi, Georgia; ^9^Faculty of Medicine, Izmir University of Economics, İzmir, Türkiye; ^10^Nofer Institute of Occupational Medicine, Lodz, Poland; ^11^International Network on Children’s Health, Environment and Safety (INCHES), Ellecom, Netherlands; ^12^WHO Collaborating Centre for Children’s Health and Environment, The University of Queensland, Brisbane, QLD, Australia; ^13^Health Services Academy, Islamabad, Pakistan; ^14^Andalusian School of Public Health, Granada, Spain; ^15^Ibs. GRANADA, Granada, Spain; ^16^IHR Strengthening Project, UKHSA, Bangkok, Thailand; ^17^ASL Foggia, Foggia, Italy; ^18^Department of Public Health and Clinical Medicine, Umeå University, Umeå, Sweden; ^19^Global Consortium on Climate and Health Education at Columbia University, New York, NY, United States; ^20^HEAL Global Research Centre, Health Research Institute, University of Canberra, Bruce, ACT, Australia; ^21^US-CDC, Atlanta, GA, United States; ^22^International Society Doctors for the Environment (ISDE), Rete Italiana Medici Sentinella per l’Ambiente (RIMSA), Arezzo, Italy

**Keywords:** environmental health, public health, ecology, professional training, education, ecological sustainability, environmental change, ecological public health

## Abstract

The global landscape of professional training in environmental health, encompassing ecological public health or environmental public health, lacks consistent global implementation for training programs for public health practitioners, clinical professionals, and individuals across various disciplines, as well as standardized curricula for undergraduates. This training gap is related to the overall lack of capacity in addressing the population impacts of the triple challenge of pollution, biodiversity loss, and climate change, impeding the worldwide transition to and development of ecological sustainability. This paper reviews existing approaches and their potential to address implementation challenges within the necessarily tight timescale. Spreading of best practice appears feasible even without substantial additional resources, through the reorientation of current practices via comprehensive multi-disciplinary training programs. By adopting international best practices of training in environmental health, the focus in training and education can shift from future decision-makers to enhancing the competencies of current professionals and their institutions.

## 1 The role of education and training in environmental public health (EPH)

### 1.1 Introduction

Human societies face a triple challenge of pollution, loss of biodiversity and climate change, that combine to produce current and expected future adverse social and health impacts (1). These trends are not restricted to Western societies but appear to accompany any drive to development in all continents and managed by the whole range of available political arrangements (2, 3). The numerous calls for a change of direction and transformation have produced limited impacts, leading to inadequate rate of change accompanied by rather frantic attempts to either deny the need to act or last ditch attempts to draw on moral foundations for change in the absence of realistic avenues for a transition to ecological sustainability of human societies. Although a response from educational specialists is expected to address the need of future generations, this would be insufficient by itself to achieve a conversion within the time period expected to be required. This paper outlines a practical element of societal response with a realistic chance of contributing to widespread adoption of relevant activities: training aiming to empower those currently employed who would wish to engage with the process of change toward ecological sustainability, associated with an institutional affirmation of legitimate remit that permits such activity as widely as possible.

The overarching questions which must be borne in mind while envisaging provision of any training or educational program are the following three. According to the overall framework mentioned in the companion paper to this (4), we are also providing our perspectives that might benefit from a comprehensive and open discussion, not only in academia but also with policymakers, businesses, trade unions, public and private health organizations, and community-based civil society groups.

1. What are some key objectives this training will achieve? Our answer: topics will be similar for all target communities, but specific objectives will vary by the target community.
2. What target communities? Our answer: (a) practitioners of public health (specialists); (b) other practitioners who have a part-time commitment to public health (PH) (e.g., family doctors, engineers, architects, environmental scientists, and several others); (c) postgraduate students who are not practicing either public health or any other profession, but intend to develop a capacity to do so.
3. What type of training? Our answer: problem-based learning directed at an appropriate level to a target audience; health services, public health, and prevention are matters that involve many disciplines and competencies (see section 4.2 and 5).

According to WHO (5), a particular attention must be paid to those working in environmental management/sustainability professions to enhance their knowledge of health/PH/EPH—focusing on the health impacts of their efforts, so also align to the EPH goals such as adoption of paradigms appropriate to the challenge (4).

Health professionals can drive social and policy change (6) as they are generally highly trusted (7) and have influence at all levels of society. With trust comes the responsibility to influence wisely and lead effectively, which requires collaborative engagement beyond individual actions (8), thus “Health professionals will be called on to engage as humble, informed, and trusted partners in the collective, boundary-crossing effort of transforming practices and structures to better sustain the health and wellbeing of all life, including our own” (9).

Such professionals should be educated and trained in the perspective of sustainable development and the green economy through public health management and risk assessment. They should be exposed to public and urban health issues, which are fundamental issues for well-being and environmental aspects at any level. Matters relating to pollution, in general, are essential for actions addressing infectious and chronic-degenerative diseases, while in urban health; the themes of strategic planning are fundamental concerning the healthiness of urban environments and possible adverse health effects.

Last but not least it is essential to integrate climate and planetary health into all general education and training of PH professionals as well as allied health professionals (10).

According to Barondess, “Professions are complex social structures derived from the guild system of specialized competencies intended to organize specialized and complex bodies of knowledge in such a way as to address both individual and societal needs. These are the basis of a social contract enfranchising the members of a profession. It makes professional knowledge central to the wellbeing of today’s society” (11). Inter-disciplinary work of health and other professionals is needed to improve delivery of ecologically sustainable societies.

The field of professional training for environmental health, whether called ecological public health or environmental public health, lacks consistent implementation globally of training programs for practitioners of public health (12), clinical medicine (13), and other disciplines, as well as educational curricula for undergraduates (14). Overall, the training effort is inadequate with reference to the triple challenge of pollution, loss of biodiversity, and climate change (15). This impairs the transition to ecological sustainability of communities globally.

The goal of the present paper is to highlight that several existing approaches and experiences represent a tangible demonstration that the most common barriers to implementation may be addressed, even in the absence of significant additional resources, by re-orientation of current practice through training of practitioners. Adopting best practice available internationally in this area, would shift the focus in training and education from the responsibilities of future decision makers and professionals, to those of those currently in post. In alignment with these objectives, Appendix A (see Supplementary materials) offers a practical context by delivering a comprehensive analysis of various field experiences conducted at both national and international levels within the sphere of education and training.

### 1.2 The role/task of EPHT and HIA

The role and task of Environmental and Public Health Tracking (EPHT) and Health Impact Assessment (HIA) is to integrate understanding, evaluation, professional profiles, and institutions (including tools). EPHT has the potential to serve as a concrete tool to “…improve the effectiveness of adaptation and mitigation strategies, assess progress toward nationally and internationally agreed targets, act as an early warning system, and hold decision-makers accountable. Indicators for inclusion in the system should be prioritized using transparent criteria, including relevance, sensitivity, sustainability, scalability, accuracy, economic viability, and consistency” (16). It has been defined as: “The ongoing collection, integration, analysis, and interpretation of data about environmental hazards, exposure to environmental hazards, human health effects potentially related to exposure to environmental hazards. It includes dissemination of information learned from these data and implementation of strategies and actions to improve and protect public health” (17).

It is an approach that helps to increase the understanding of environmental public health and global health, improve comparability of risks between different areas of the world, and enable transparency and trust among citizens, institutions and the private sector, and inform preventive decision-making.

Environmental and Public Health Tracking is also a helpful tool for strengthening the established Driving Forces, Pressures, State, Exposures, Health Effects and Actions (DPSEEA) framework (18). EPHT promotes a systematic integration of the DPSEEA components above-mentioned, taking environmental and health parameters into account. For each stage of this scheme, one can design and/or evaluate indicators to measure the impact of interventions at each level (19). An example from Cuba (Table 1) shows that an ecological approach to environmental health has been applied decades ago, and the DPSEEA framework may clarify requirements of public health surveillance/EPHT in relation to specific interventions/actions (20).

**Table 1:** Examples of environmental health indicators within the DPSEEA framework.

Concern: causal chain		Housing-related indicators	Microbiological indicators (water contamination)
Driving force	Type of development or human activities	Migration	Sewage generation
		Life conditions index	Water pipe deterioration (aided by embargo)
Pressure	Amount or size of production	Housing quality	Amount of waste produced
		Water supply	Amount of untreated effluent
	Emissions	Sanitation facilities	% of broken sewage lines
		Liquid and solid wastes	
State	Environmental effects	Microbiological contaminants	Coliforms in water, food
		Standing water (vector breeding)	
		Pests, rodents, and pathogenic organisms	
Exposure	Human exposure	Proportion of households/people exposed to pests, rodents, and vermin	Estimated exposure to contaminated food/water
			Serum analysis for Hepatitis and typhoid
	Dose	Parasites in stool	Feces for cholera, Shigella
Effects	Early effects	Diarrhea, fever	Diarrhea, fever, and nausea
		Gastrointestinal diseases, parasitic	Cholera, Hepatitis A, typhoid, dysentery, and gastroenteritis
	Late effects	Leptospirosis	Death from dehydration
	Death due to:	Death from dehydration	
Actions	Interventions can be identified and associated with each level of DPSEEA framework		

Source: Modified from Yassi (20).

Environmental and Public Health Tracking aims to promote a resilient society by analyzing complex datasets, addressing different audiences, and supporting environmental health messaging tailored to each audience:

- The public: information to support individual changes in attitudes and collective actions.
- Professionals and stakeholders: tailored information to health professionals, land planners, urban planners, environmental managers, policy makers, and researchers.
- Decision-makers: integrated health and environmental information to inform decisions and create opportunities to reduce the multiplicative impacts of rapid urbanization, globalization, and climate/social/economic change (21).

Such general and generic categories also include resource managers, planners, economists, conservationists, indigenous and locally impacted communities, community developers, and other essential stakeholders. They are all strategically important, taking into proper account the dynamics that interrelate the two central issues on how population health may be improved: individual behavior and social and economic factors (22, 23). A conceptual framework to integrate economic and other dimensions for ecological public health facilitates identification of appropriate interventions (24).

The EPHT approach strives to achieve its vision of “*Healthy Informed Communities*” by empowering environmental and public health practitioners, healthcare providers, community members, policymakers, and others to make information-driven decisions that affect health while maintaining appropriate data protection measures (17). Several technological applications have become available (25, 26) or present considerable promise (27) that can support EPHT operations by a range of stakeholders.

In this context, Health Impact Assessment (HIA) has been proposed as the combination of methods to support Health in All Policies (HiAP) implementation by providing scientific evidence on the positive and negative effects that any new program, project or proposal may have on health and health equity.

The availability of evidence is often perceived as consistently insufficient when making decisions related to various aspects of public health, encompassing emerging issues and interventions’ risk–benefit assessments. In practical terms, decisions must align with current resources and tangible budgets, prompting consideration of an alternative analytical approach known as “decision analysis.” This approach operates on the premise that decisions are made amidst incomplete knowledge, and not all pertinent facts are accessible for prevention assessments. In essence, the strategy for prevention involves choosing between interventions, each with associated costs and benefits. Comparing two alternative choices, A and B, directed at the same prevention goal allows the establishment of a ranking based on their cost–benefit ratios. This approach facilitates the comparison of knowledge supporting different choices in terms of their overall health impact.

The decision analysis employs multi-criteria decision analysis (MCDA), a methodology applicable to risk prioritization, especially in national decision-making. While other methods, such as cost–benefit analysis (CBA) and cost-effectiveness analysis (CEA), focus on economic considerations, MCDA accommodates a broader range of criteria, including qualitative and quantitative evidence. As we navigate global challenges, the call for “new coalitions and partnerships across many disciplines” is imperative. The ultimate goal is a comprehensive integration within a “planetary” framework, addressing environmental and public health outcomes. This perspective underscores the necessity for a global outlook in delivering ambitious objectives (28–30).

In summary, EPHT is an instrument that can support the cross-sectoral integration of information to assist decision-making in support of the utmost ambitions for global and planetary health outcome.

The core infrastructure of EPHT within national public health agencies can deliver both capacity to support ongoing concerns on hazardous pollutants and chemicals in drinking water, land, food and air and new perspectives on the central value of ecological and social factors in affecting health and wellbeing in the course of multiple transitions currently experienced by society (21). The latter perspectives are explored initially in research partnerships between national public health agencies operating EPHT programs and academic or other relevant research institutions.

This process means EPHT may support mainstream public health operations and partnerships in transitioning toward more appropriate consideration of ecological and social factors in health protection and health improvement activities (31).

### 1.3 Experiences of integrated training across the world

We have worked with several professional networks of which we are part to review experiences in training and education of professionals that have a role in advising decision makers about human activities with known impacts on public health, identifying those training activities that aimed at re-orientation of a human activity toward ecological sustainability. We have extracted from a purposeful selection of experiences those elements that could contribute to recommendations for general application in comparable contexts.

Core elements of what constitutes good public health practice have a strong focus in a curriculum, so that public health trainees will have an opportunity to demonstrate in their actual service both the confidence and competence necessary to go on to develop increasing levels of expertise in their subsequent, more specialized professional practice. Further details of country-level as well as supra-national experiences have been collated and are presented in Appendix A. There are cultural, disciplinary, institutional and economic types of obstacles in developing, running and completing training programs that address the issues highlighted. The fact that several experiences have already been completed means that objections to this approach can be overcome in practice, giving rise to an expectation that such practices can be extended each within its milieu and beyond.

Public health trainees and others involved in learning about their role as a public health agent are expected not only to know about good public health practice and show they can do it or apply it in a protected setting, but, over the length of the training program, to undertake and do their daily work with required levels of knowledge and understanding and at increasing levels of complexity. The Miller Triangle has been used to illustrate the three phases of any health training program, moving from learning through formal study (phase 1), to learning from service experience and increasingly complex service work (phase 2), to demonstration of integrated practice of complex competencies (phase 3) (32). This approach has been widely used for the assessment of professionals in health care, clinical and scientific/technical (33), and has been extended to application of ecological determinants of health by public health professionals (34).

**Figure 1:**
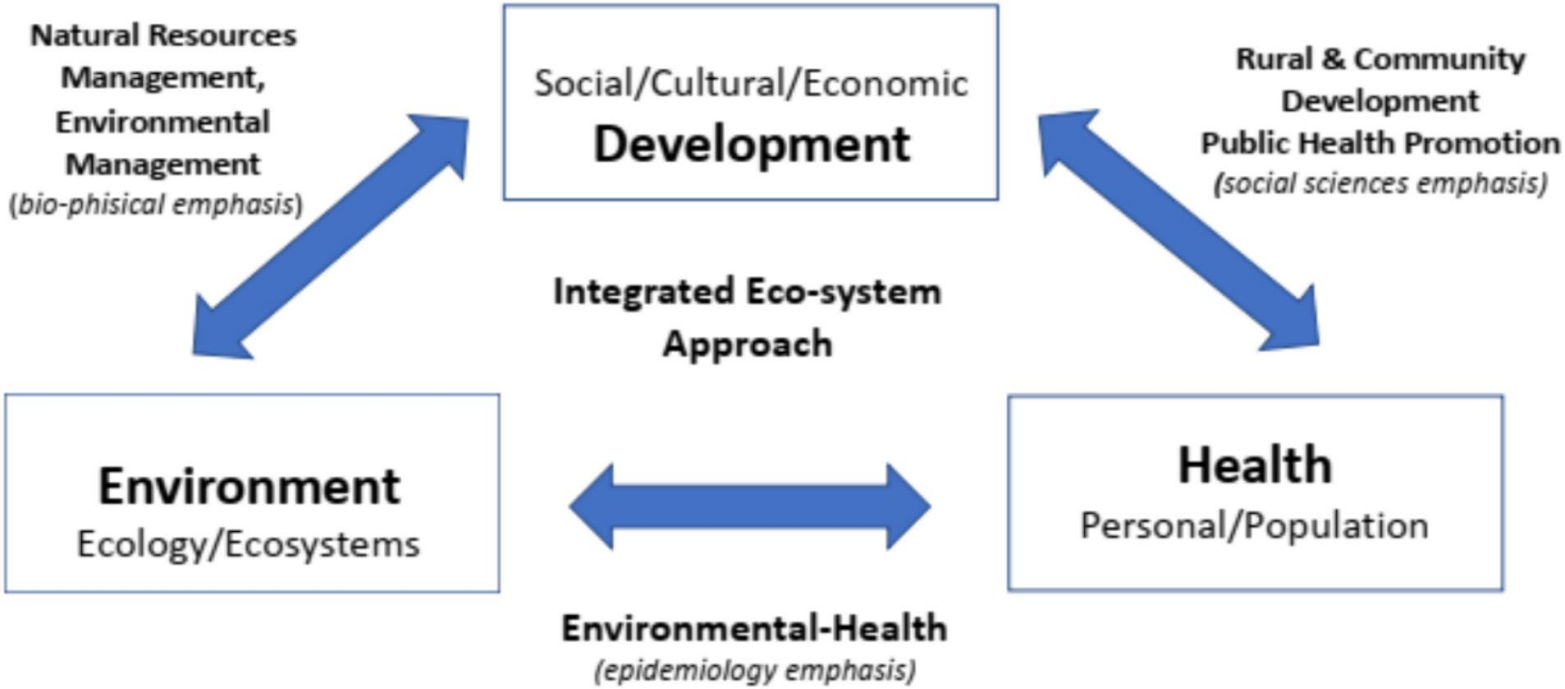
The “HEAD” (Health, Environment, and Development) triangle: links between different disciplinary territories. Source: Parkes (35) (modified).

To achieve the desired social and health benefit, a participatory approach in design and interpretation of EPHT or surveys or other activity intended to support or implement public health interventions has been recognized as crucial (35). Three broad disciplinary areas are thus put in relation with each other: natural/biophysical sciences, epidemiology, and social sciences (Figure 1). The shared goal of ecological sustainability of a community may not be achieved when excessive focus is placed on only one or two of these.

The educational theories that have underpinned effective integrated training often include problem-based learning, using engaging tasks or problems as a starting point for self-directed and self-regulated learning, thus encouraging trainees to express and share with others their skills, experience and knowledge (36). Training methods can then be broadly interpreted and include role play (37), field visits, leading to experiential and masterly learning, participatory and deep learning. Case studies provide an effective learning setting to incorporate different learning models with the purpose of developing a set of practice-oriented skills, via mobilization of cognitive and psychomotor participants’ skills, values, attitudes, and feelings (38).

## 2 Proposals for training and education in environmental public health

### 2.1 Preparing the current workforce (retraining and continuous professional development)

#### 2.1.1 Overall approach

The imperative to fortify environmental health curricula arises from global challenges encompassing chemical exposure, ecological shifts, and climate change, posing intertwined social and health impacts (39). Seven pivotal developments in environmental public health emerge in response to these challenges: occupational and environmental health, political ecology of health, environmental justice, eco-health, One Health, ecological public health, and planetary health (28). While each development holds value, certain limitations exist. For instance, the One Health framework, though addressing the triple challenge, tends to emphasize human and animal health over environmental drivers. Recognizing the urgency of the environmental public health task, we propose a pragmatic approach—integrating environmental and ecological considerations into existing curricula rather than creating separate ones. This approach necessitates a philosophical shift in perspective but requires only a minor adjustment in professional skills and competences (40). A framework for curricula that we propose could help standardize training of any practitioner in developing competences expected to be beneficial to EPH is presented in Table 2, however based on the criteria and recommendations in this paper other frameworks may be equally valid. Several specific topics and case studies have been identified for each of the themes listed, but these would differ by type of practitioner and cultural/disciplinary/institutional context.

**Table 2:** Elements of proposed curriculum for training to build capacity in Environmental Public Health.

General concept area	Themes
*Environmental public health functions* with specific goals to be defined and achieved in partnerships with local communities, health and social care services, and professional/scientific societies	*Intervention-building approaches (including cost–benefit analyses)*
	*Evidence reviews for policy/decision makers*
	*Risk assessment, risk management, risk communication*
	*Environmental public health tracking/Health impact assessment or analysis*
	*Response to events and preparedness*
*Population thinking:* “*a mode of conceptualizing issues for a whole group of people defined in a certain way*.”	*Ethical, cultural, social, policy aspects*
	*Defining populations over time*
	*Measurement (including exposure science)*
*Group Comparison:* “*contrasting what is observed in the presence of activity/exposure to what would have occurred had the group of interest not been exposed to the postulated cause*.”	*Study designs*
	*Use of modern statistical analysis methods*
	*Causal inference*

Given the urgency, focusing on training the current workforce becomes a priority. This calls for a recognition that a new remit within existing domains can be admitted professionally and legally. The experiences shared in this paper aim to encourage other countries to review and approve similar curricula, seeking legal recognition for professional practice. Acknowledging the diverse roles within this broader public health field—public health specialists, those indirectly involved, and professionals aware of public health implications—requires separate curricula tailored to their specific needs.

As illustration of such wide range of roles, an example will be given for each of these three groups.

#### 2.1.2 Public health practitioners

Typical aspects of the role of a public health specialist are: (i) Population thinking (in relation to exposure, drivers etc.); (ii) Interpretation of health data (in relation to eco-social and other factors); (iii) Identification of causes susceptible of primordial prevention. Professional activity in public health has been shaped by challenges that point to the need to adopt a population perspective in assessment and management of multiple issues. Communicable and non-communicable disease each have been addressed by development of a set of competences adopted by most countries. Recognition of the environmental drivers, pressures, states, and precursors of each type of disease facilitates matching with appropriate interventions available at each level to minimize and prevent disease.

Therefore, a public health specialist working on environmental public health could be trained in each country, by provision of a curriculum integrated with existing professional competencies and remits. Such curriculum could be broad to include competencies to address pollutants (41), and ecological aspects (42), as well as climate aspects (43) in relation to health, as needed to address co-existing drivers across these themes. Overall, the lessons from countries and settings reviewed here, point to the feasibility of such enterprise elsewhere. A committee comprising staff from national public health and professional societies of practitioners could complete the process of establishing a curriculum inspired by experience of others, but tailored to the local needs and priorities (Table 3).

**Table 3:** Several levels of depth at which the training of practitioners in public health may be appropriate.

Audience within public health	Level of training	Mode of training
A. Decision makers in public health would have remit for district-level budget and operational decisions.	A few hours	Online 100%
B. General public health workforce may require an overview of the topics and access to appropriate subspecialists, able to chair working groups on specific themes, brief decision makers.	A few days	Online 50%
		Face to face 50%
C. Health protection specialists, who have an ongoing remit for preparedness and response to events, public health surveillance, and general capacity to review evidence.	A few months	Online 30%
		Face to face 70%
D. Sub-specialists who have dedicated a substantial proportion of their practice in public health to environmental health themes, possibly focused on either pollution, one health, or climate-related issues. May require sub-specialty registration with General Medical Council in environmental public health.	A few years	Online 10%
		Face to face 90%

#### 2.1.3 Health care workers

The importance of the health care workers has been paramount all around the world even before COVID-19 pandemic. Societies are aging, health spending is rising in response to more complex health needs. The rapid spread of COVID-19 added complexity but provided essential lessons, which should be considered either in terms of resources to be allocated or in terms of the systematic approach, which should be comprehensively implemented in Public Health care.

Also connecting Primary Health Care (PHC) and occupational health (OH) is critical for better prevention of chronic conditions (such as musculoskeletal or mental health disorders) that lead to absenteeism or early departure from the labor force (44). Initiatives for better protecting workers’ physical and mental health could include raising awareness among managers, improving the physical working environment, humanizing social relations at work, and offering programs dedicated to encouraging disabled people to return to work. Equally important is the health surveillance of workers, through assessing and responding to mental stress and physical strain at work.

As such it is essential to remember that only a real integration of PHC, Prevention and Hospital care could create the condition for a multidisciplinary workforce which play a vital role in recognizing and managing the environmental and social factors that affect community health.

Health systems can participate in this movement by adopting two critical roles: working as “anchor institutions” to support local problem-solving efforts and serving as partners in innovative approaches to safeguarding community mental health.

In addition, PHC are the frontline of community wellbeing, and therefore can have an important role in community building, social cohesion, and resilience. COVID-19 emergence highlights the need to place such strengths at the forefront of any emergency plans, as these can help align solutions that the current and more general climate crisis demands with a compelling vision of local well-being and participation (Table 4).

**Table 4:** Several levels of depth at which the training of health care practitioners may be appropriate.

Audience within health care	Level of training	Mode of training
A. Decision makers in health care would have remit for district-level budget and operational decisions.	A few hours	Online 100%
B. General health practitioner (medical or nurse or physiotherapy or other), may require an overview of the topics and access to appropriate subspecialists, able to chair working groups on specific themes, brief decision makers.	A few days	Online 50%
		Face to face 50%
C. GPs and Pediatricians need to implement a careful attitude in registering data of their patients’ health status, occupation, life style, socio economic status, ambient and home environmental conditions.	A few days	Online 50%
		Face to face 50%
D. GPs and Pediatricians who wish to adopt availability to communication with individuals and communities in their professional practice.	A few months	Online 30%
		Face to face 70%
E. Dedicated health practitioners, who have an ongoing remit for providing public support through networking, preparedness, and response to events.	A few months	Online 30%
		Face to face 70%
F. Sub-specialists who have dedicated a substantial proportion of their practice in health care to transforming health care systems toward ecological sustainability.	A few years	Online 10%
		Face to face 90%

The key tasks for health care workers to contribute to ecological public health are:

- Encouragement of decisions toward health care systems that maximize resilience to disasters and are ecologically sustainable.
- Advancement towards a resilient approach to address global threats at the local level.
- Integration or at least networking among all facets of health practice (i.e., PHC, Prevention, and Hospital).

#### 2.1.4 All others (using examples of architects and town and country planners)

There has been a recognition of the need to consider social and health impacts as an indicator of the ecological sustainability of activities in sectors different from health; for example, town and country planning and built environment, and also agriculture and forestry, transport, education, military and civil protection (see Table 3) (45).

Although the example of town and country planning and related built environment topics is described here, a similar graded approach is promoted in all the sectors (Table 5).

**Table 5:** Several levels of depth at which the training of town and country planners may be appropriate.

Audience within town and country planning/architecture	Level of training required	Mode of training
A. Decision makers in town and country planning would have remit for district-level budget and operational decisions.	A few hours	Online 100%
B. General workforce (architects, surveyors, town and country practitioner), may require an overview of the topics and access to appropriate subspecialists, able to chair working groups on specific themes, brief decision makers.	A few days	Online 50%
		Face to face 50%
C. Dedicated workforce, who have an ongoing remit for advocacy, networking, preparedness and response to events.	A few months	Online 30%
		Face to face 70%
D. Sub-specialists who have dedicated a substantial proportion of their practice in town and country planning to transforming communities toward ecological sustainability.	A few years	Online 10%
		Face to face 90%

### 2.2 Preparing the future workforce

#### 2.2.1 Overall approach

Based on available frameworks that combine global and local aspects of environmental health (46, 47), curricula already exist for diploma and degree education across all human roles, and there are already numerous examples of environmental health elements having been integrated in such programs (Table 6).

**Table 6:** Four domains of knowledge required for environmental public health education in climate-related facts.

Level	Learning objectives
Factual knowledge	*Universal basics*: social and environmental determinants of health, psychology of suffering, community response, and behavioral change.
	*Climate-related facts:* health co-benefits of climate action, sustainability of health factor.
Conceptual knowledge	*Universal foundation*: equity, vulnerability, precautionary principle.
	*Climate-related facts:* sustainability, “eco-health,” planetary boundaries.
Skill-related knowledge	Universal foundation: evidence-based medicine, health education, science communication, collaboration, and system thinking.
	*Climate-related facts:* clinical diagnosis and management of climate-associated diseases.
Emotional competencies	*Universal foundation:* importance of medical education to society at large, benefits of multidisciplinary collaboration.
	*Emotional competencies related to climate-related facts:* appreciation of the complicated relationship between equity, sustainability, and health.

Source: Boekels (48) (modified).

Several barriers to implementation have been identified: (i) lack of knowledgeable teachers of sustainable health systems; (ii) lack of space in the curriculum; (iii) uncertainty of location in the curriculum; (iv) need for learning resources; (v) difficulty in assessing learning; and (vi) emotional impact needing resilience (49). Also drivers and enablers emerge: (i) demand from students; (ii) the move to include sustainability in higher education; (iii) a new legitimacy through mandate of professional bodies; (iv) leadership from other stakeholders; and (v) several sources of support and resources (47).

Having identified best practice from several countries and areas where such initiatives have been conducted, points to the feasibility of rolling these programs to other areas. The scale effect of widespread adoption of such initiatives may spill over beyond the direct awareness of those undertaking such courses and generate sufficient momentum to provide motivation to current decision makers to accelerate the overall trend toward ecological sustainability of human society.

Future success of environmental public health rests on joint action of three groups: (1) public health students; (2) health care students (clinical and other); and (3) other disciplines required for education of all those who should be aware of the implications of their activity on development of ecologically sustainable communities. Recognizing this, educational curricula for diplomas and university qualifications will be required to address needs of such disparate roles. As illustration of such wide range of roles, an example will be given for each the groups.

#### 2.2.2 Public health education

It is common practice that public health practitioners are not training for environmental health topics. For example, in the United States, MPH degrees do not include training in climate change and health issues (50). A combination of competence in specialist disciplines (natural sciences, toxicology, environmental epidemiology, risk assessment, and environmental public health) and general public health skills (management, research, and teaching) was identified in United Kingdom as a requirement of environmental public health education (51). A broad program based on these dimensions, with added inclusion of disciplines relevant to climate and other environmental change, appears as feasible for education of public health students. With focus on climate change, we support a broad six-domain competency framework consisting of (1) climate and environment sciences, (2) drivers of climate change, (3) evidence, projections, and assessments, (4) iterative risk management, (5) mitigation, adaptation and health co-benefits, and (6) collective strategies-harnessing international/regional/local agreements and frameworks (52).

#### 2.2.3 Health care education

Five core domains have been identified by the Global Consortium on Climate and Health Education (GCCHE), with over 300 health professional member institutions from 56 countries: (i) Knowledge and analytic skills; (ii) collaboration and communication; (iii) policy; (iv) public health practice; and (v) clinical practice (53). Three areas have been recognized for learning objectives by the Center of Sustainable Healthcare: (i) Describe how the environment and human health interact at different levels; (ii) Acquire the knowledge and skills needed for more sustainable health care systems; and (iii) Discuss how a physician’s duty to protect and maintain human health is affected by the local and global environment (54).

Three areas that deserve attention when developing curricula in this sector are: (i) options for integration of climate change content into existing courses and curricula; (ii) available range of teaching methods such as problem-based learning; and (iii) options for issuing certificates of achievement in the field of environmental health/climate (46).

#### 2.2.4 Education in other disciplines

In almost all of United States universities, climate change was taught in graduate programs (sustainability, urban affairs, geography, and geosciences), but was not cross-listed for the public health program. The implication is that lack of specific training will be detrimental in designing mitigation or adaption approaches for agencies and organizations (50). To create healthy communities, a wider use of accepted science needs to be applied to education of future practitioners in courses (different from health) about climate change and potential strategies for interventions effective to increase resilience of communities.

## 3 Discussion and suggested approaches

### 3.1 Discussion

This paper explores the feasibility of providing comprehensive training for environmental public health practitioners and educating students across disciplines to prepare them for addressing the ecological sustainability challenges confronting communities dealing with pollution, biodiversity loss, and climate change, alongside social, economic and health challenges. Our proposal promotes tailored training and education for specific audiences, varying according to their existing roles. While the documented successful experiences impact a relatively small segment of the workforce, it is pragmatic to plan the expansion of existing initiatives to areas currently lacking such training and practices, in different context, geography and disciplinary range.

Examples that we are referring to (Appendix A) including developments in the areas of citizenship, history, technology, and natural sciences curricula have already established a field of education that is highly innovative and valuable for social resilience in the face of current challenges. These curricula instill confidence in applying concepts, skills, and established competencies among professionals from diverse backgrounds, including specialists in public health with expertise in biology, physics, chemistry, sociology, anthropology, nursing, epidemiology, nutrition, or other scientific fields. Despite their efficacy, these experiences encounter limited acknowledgment for registration within legally defined organizations that authorize specialist-level practice, hindering their geographical distribution across countries and continents.

Limitations of our review include the reliance on practitioner perspectives, with minimal reliance on systematic reviews. However, the approaches gathered here cover a wide range of geography, cultures, and practitioner perspectives, and they consistently converge on essential domains. Despite insufficient evaluation of training translation into interventions and public health benefits, identified barriers have been overcome in many cases, providing a foundation for extending these experiences globally. Attention to the registration of trained practitioners in legally recognized roles is crucial, especially outside Europe, as it could form a social infrastructure supporting community resilience within the broad public health economy. Acknowledging differences in capacities and infrastructure across communities and countries, our proposal leverages existing capacities and public health infrastructure globally, using training as a catalyst for strengthening and creating new capacities in environmental public health. Existing international experiences indicate the potential for widespread adoption, emphasizing the transformative impact of developing strong EPH capacity. The overall ambition is that all communities will: first, recognize the value of isolated inter-disciplinary experiences in their midst or elsewhere; second, that such awareness will be extended and translated to acceptability by professional societies in terms of accreditation of such experiences; and third, that any community wishing to initiate their own program for training would be facilitated by networks highlighting available standards and promoting best practice.

We have presented several examples of successful practice in EPH, but many do not include evidence on the persistence of any activity beyond the life of a project or program. Based on the experiences reviewed, competencies required to build integrative practice across professions have been identified and could be further established and promoted. Alongside the ecological sustainability of societies, these proposals need to be sustainable also for the life of practitioners. Those who have undertaken professional training in EPH could be followed up to see if they are employed in these roles, embedded in relevant milieus, for how long and with what impact.

Legal recognition of multi-disciplinary experiences conducted as part of an overall acceptance of a professional qualification has been achieved in some countries (see Appendix), further extension may be facilitated by appreciation that a few months of training embedded within a different disciplinary program makes a substantial difference to the overall competence reached for EPH. The WHO could support this by including EPH as part of their definition of “essential health service.”

Also, standards and key performance indicators for professional training could be adapted by professional societies, who could provide a feedback mechanism for practitioners in training to contribute to ongoing review, evaluation and adaptation of training programs.

This review did not differentiate between global and local applications of the proposed EPH practices and how they can be tailored to meet specific needs. We have assembled examples along a range of scopes and themes, and these include many local but also several global applications such as contribution to global UN processes. Some practitioners have noted that achievement in local EPH may be a way to gain confidence and lead to increased validity of proposals for input into global dimensions of EPH.

Research agendas that would contribute to the proposed program would be established ideally in collaborations between innovative multi-disciplinary professional training schools and centers of academic excellence. The motivation to achieve impacts on public health by new synthesis of knowledge and practice would be shared by individuals and institutions promoting such consortia. A key dimension of success of a comprehensive research program would be a balance between (i) space for most innovative interventions such as social prototypes with experimental characteristics, and (ii) promotion of interventions inspired by standards based on replicated effectiveness and supported by competency acquired as part of a training program for practitioners (such as by professional doctorates). Ideally, this balance would be mirrored by a flexibility of professional training programs allowing reaching standard competencies with the widest possible field of application ranging from agroforestry, built environment and social care services.

### 3.2 Suggested approaches

1. To enhance broader public awareness and education, it is crucial to invest in increasing citizens’ literacy regarding the conceptual and practical foundations of environmental public health. This literacy is essential for fostering community resilience.
2. In response to the acknowledged challenges in environmental public health and with the objective of fortifying the EPH functions across society, institutions overseeing the training of professionals and education at both undergraduate and postgraduate levels should consider it imperative to adapt existing curricula and develop new ones. These curricula should encompass comprehensive environmental public health knowledge, hands-on experiences, and the instillation of innovative ideas.
3. To enhance the robustness and significance of current elements within professional training curricula related to environmental change, encompassing topics like climate change, environmental pollution, and biodiversity loss. A key step of the reform initiative should concentrate on sectors already involved in public health matters, including public health practitioners, the healthcare workforce, and other professionals engaged in public health. The innovation we propose would allow that the EPH training activities are based on practice and led by the practitioners as agents for change. A key step will be the acceptance of multi-disciplinary training periods to the point of legal recognition for practice at basic, specialist or sub-specialist level.
4. In parallel, similar reform initiatives to develop, standardize curricula for professionals and practitioners in sectors different from health, with a systematic mapping of the social and health benefit of their activities, are needed by professional societies.
5. National ministries overseeing diverse sectors, such as environment, industry, energy, transport, agriculture, housing, social care, and development, should collaborate with the ministry responsible for higher and professional education, along with related budget-holding agencies. This collaboration may be most valuable when strengthening and enhancing the relevance of curricula for professionals in sectors traditionally distinct from organized public health. This includes individuals like engineers, architects, town and country planners, agronomists, and forestry managers. High-level recognition as suggested above such as WHO considering EPH an “*Essential Health Service*”—and similar recognition by frameworks that inform country/regional allocation for resources such as International Health Regulations (IHR).
6. A particular care must be paid to improve processes for consultation about programs and projects with expected social impacts, by appropriate governance arrangements and communication skills such as listening, speaking, observing and empathizing and also the capability to persuade others on a topic without using force or compulsion while respecting their viewpoints.

## 4 Conclusion

1. Environmental aspects of health protection and promotion have emerged as crucial public health dimensions over the last 50 years. There is an increasing awareness of the urgency to review and reform education and training. An initial survey of the range of roles that could be identified as already engaged in environmental public health indicates that a few identifiable groups of students and practitioners could be involved in developing the capacity and capability to contribute to society’s resilience in the face of current environmental epochal challenges.
2. There have been valuable ideas and experiences relevant to fundamental education in concepts and awareness of environmental public health practical skills at undergraduate and professional or postgraduate levels.
3. Valuable insights have emerged for postgraduate and professional curricula, benefiting the training of natural scientists, social scientists, health professionals, and epidemiologists already employed in functions of environmental public health within the formal health sector. Harmonizing and disseminating best practices across different countries and continents present several hurdles. Social organization (relation between academia and other organizations), pragmatisms, culture, socioeconomic context will affect how the training is organized and implemented.
4. Insightful ideas and experiences have surfaced regarding postgraduate and professional curricula aimed at training individuals in sectors traditionally distinct from organized public health in environmental public health. These curricula foster confidence in applying concepts, skills, and established competencies among professionals such as engineers, architects, town and country planners (both urban and rural), agronomists, and forestry managers, despite their lack of professional training in public health (also referred to as hygiene).
5. Nonetheless, these experiences encounter limited recognition for registration with legally-defined organizations such as professional and governmental agencies that authorize specialist-level practice, impeding their geographical distribution across diverse countries and continents.
6. Processes for consultation with all stakeholders including representatives of communities affected by such activities and their ecological impacts are often lacking or not implemented. Effective EPH practice depends crucially on inclusive nature of the processes used for designing, promoting, implementing, and evaluating programs and projects whose ecological aspects may affect public health.

## Author contributions

GL: Conceptualization, Formal analysis, Supervision, Writing – original draft, Writing – review & editing. AZ: Conceptualization, Formal analysis, Funding acquisition, Resources, Supervision, Writing – original draft, Writing – review & editing. MA: Writing – review & editing. CB: Writing – review & editing. HC: Writing – review & editing. RD-D: Writing – review & editing. RE: Conceptualization, Writing – review & editing. NG: Writing – review & editing. OG: Writing – original draft, Writing – review & editing. WH: Writing – review & editing. PJ: Conceptualization, Writing – review & editing. EK: Writing – original draft, Writing – review & editing. PM-O: Writing – review & editing. JP: Writing – original draft, Writing – review & editing. PH: Writing – review & editing. ER: Writing – review & editing. MS: Writing – review & editing. JS: Writing – original draft, Writing – review & editing. CS: Writing – original draft, Writing – review & editing. SV: Writing – original draft, Writing – review & editing. FY: Writing – review & editing. PL: Conceptualization, Formal analysis, Supervision, Writing – original draft, Writing – review & editing.
