# Supplementary material for: Building competency to deal with environmental health challenges: experiences and a proposal
Source: Front Public Health. 2024 Nov 18;12:1373530. doi: 10.3389/fpubh.2024.1373530 (PMC11627219; doi:10.3389/fpubh.2024.1373530)
Supplement: Supplementary file 1 [file Data_Sheet_1.pdf]

Appendix A : Experiences and lessons learnt

Summary

1. Country-level 2

1.1. Australia 2

1.2. Belgium 2

1.3. Canada 4

1.4. Some lessons from France 4

1.5. Georgia 4

1.6. Italy 5

1.7. Poland 5

1.8. Turkey 6

1.9. The UK 7

1.10. United States of America 8

1.11. Zambia 9

2. Supranational experiences and lessons learnt 10

2.1. International Network on Public Health and Environment Tracking – INPHET 10

2.2. Lessons across Europe 10

2.3. Lessons from World Health Organisation WHO 11

2.4. Environmental health in International Health Regulation implementation 11

References 13

1. Country-level

1.1. Australia

Experience in Australia indicates the importance of rapid evidence assessments or rapid health impact assessments to support climate and health policy development in a timely manner. Traditional research studies usually take longer (years rather than months) than policy initiatives, so the need was recognised to develop rapid assessment methodologies and skills that can underpin such policies. For example, the implementation of the Australia National Health and Climate Strategy, launched in December 2023, was supported and enhanced by rapid reviews of the evidence within a framework of consultation with a wide range of stakeholders on topics including measurement, built environment infrastructure, travel and transport, prevention and optimising models of care, waste, supply chain, as well as health and wellbeing. [1] A specific example concerns the rapid health impact assessment on wood heater smoke, for which evidence was produced that was inspired by the opportunity to support a regional policy aiming to phase out wood heaters in the Australian Capital Territory.[2]

Indigenous communities shoulder a disproportionate burden of ill health compounded by climate change. Indigenous community engagement has been increasingly recognised as essential for success of environmental health interventions in Australia. In that continent, the oldest surviving cultures have adapted their ecological knowledge over millennia and across climatic ages. European colonization has severely curtailed Indigenous peoples' ability to adjust to climate change. An effective response to the climate crisis requires decolonizing processes to reform our relationship with the planet. From an Australian Indigenous perspective, precursors for a self-determined and healthier future are justice, culture, and relationships. [3] These values deserve wide recognition in the political , policy-making, and professional structures of each country including Australia.

1.2. Belgium

In line with the PHEEDUNET study, [4, 5, 6] a study was launched within the NEHAP to identify the existing curricula related to environmental health and environmental medicine, in Belgium. The inventory of trainings or related trainings in public health, environmental sciences, environmental specialties, occupational medicine, and medicine, identified 23 programs and trainings, 55 courses at 5 universities and 2 high schools with environment & health primers, in 2011. [7] Universities and Superior schools are the main producers of educational programs in Belgium.

A specialised master in environmental medicine

No trainings on environmental medicine had yet been identified at Belgium universities in 2011. Since the publication of the inventory, a university diploma in environmental medicine has been launched by one of the universities (Université libre de Bruxelles) in 2019 but had been slowed down by the covid pandemics. The objective is to experiment specific training based on cases, scenario's, and good practices for clinicians (medical professionals, nurses and mid-wife's) for them to search for

environmental exposures to explain the diagnosis and identify treatments on the source of exposure rather than treating the symptoms.

Specialised master (environmental public health)

No specialised master as a full curriculum has been identified in Belgium whether for clinicians or environment specialists. Long-life learning programs supply with specific subjects (see Life Long Learning section)

Master in environmental public health

Four Belgian universities have been identified to provide a master with an increased insight in health and environment:

The “Master in *de biomedische wetenschappen: milieu en gezondheidswetenschappen*” (120 ECTS)[a] at the University of Antwerp was established at the end of the 1990s The biomedical sciences field of study is a multidisciplinary training with teachers from the faculties of Pharmaceutical, Biomedical and Veterinary Sciences, Medicine and Sciences. At the same time a module ‘man & environment’ of 4 ECTS was made compulsory in the bachelor of Medicine until 2012.

The “Master in de biomedische wetenschappen: Milieu en gezondheid” (120 ECTS) at the University of Hasselt offers a course which deals with biomedical, biological, toxicological and chemical aspects. In addition, the focus is on the social dimension of environmental issues. This master exists since 2003.

The « Master en sciences de la santé publique - option Promotion de la santé et environnement » (120 ETCS) exists since 2003, at the University of Liège and provides the students with knowledge to draw up, implement and assess health effects related to environmental determinants and develop qualitative and/or quantitative research projects, to manage and analyze databases and use health information when taking decisions. The curriculum is not only focused on acquiring clinical skills either, as this is addressed in medicine and pharmacy, but prepares so much for technological or scientific research in a clinical context.

The Université Libre de Bruxelles offers the "Master en sciences de la santé publique à finalité Santé Environnementale" program since 2007. This 120 ECTS master focuses on environmental health, training students with diverse backgrounds, including healthcare professionals. The goal is to address environmental health similarly to how general practitioners approach individual health. Graduates gain skills in assessing health effects related to environmental determinants, conducting research projects, and using health and environmental information for decision-making, contributing to primordial prevention. The curriculum covers toxicology, epidemiology, risk assessment, and a systemic approach.

Specialised courses in environmental health integrated into Bachelor and Master curricula

---

[a] ECTS stands for European Credit Transfer System and provides a way of measuring and comparing learning achievements, and transferring them from one institution to another. One ECTS is equal to 28 hours of study. Bachelor's and Master's degree course subjects have a study load of either 5 ECTS or 10 ECTS, which is equal to 140 (5 x 28 hours) and 280 (10 x 28 hours) study load hours, respectively. The total study load for a three-year Bachelor's degree course is 180 ECTS (3 x 60 ECTS).

Specific modules and courses were embedded in various university and superior schools' curricula with more or less emphasis. Since the survey in 2011, new courses have been introduced and re-enforced within most curricula for health professionals (ref). Specific modules are embedded in most master and advanced master trainings, such as medicine, occupational medicine, public health, as generic or specialised in health management, in epidemiology, in health promotion, nursing, mid-wife, advanced nursing practices, biomedical sciences. On the other hand, health modules have been introduced in the master in environment science and environmental management.

Long life education on environmental health (Long Life Learning, LLL)

- For physicians and medical professionals
- For public health professionals
- For other non-medical and non-public health professionals

There are a few other organizations that provide trainings for the health care sector. Professional associations are the main providers of continuous professional training for their related group of health professionals and a few small-scale initiatives in the field of environment and health have been proposed. But no training in the field of environmental health or environmental medicine with a structural continuous format has been identified. Several one-shot trainings) are organized by the scientific society for medicine (SSMG).

1.3. Canada

The Canadian Community of Practice in Ecosystem Approaches to Health (CoPEH-Canada) have developed a programme of land-based, transdisciplinary, learner-centred, transformative learning and training. [8] In 2008–15 the programme delivered an 11 day Workshop and summer field school on ecosystem approaches, from 2016 a hybrid, multisite, field course and webinar series on ecosystem approaches to health to health has been developed. The objective has been to apply ecosystem approaches to health principles to environment health-society issues, while building a community of practice.

Land-based healing is promoted by Indigenous practitioners with cultural knowledge relevant to harvesting, education, ceremony, recreation and cultural-based counselling as component of integrative practice for health and wellbeing. Land-based healing taught for millennia, and its value for ecological public health has been receiving increasing recognition in Canada. [9]

1.4. Some lessons from France

In France there are many initiatives to inform/form about the health risks of environmental determinants. Among them, there is a EU-funded training program on air pollution and health, Airducation [10], towards different target audiences, among them health professionals. A big effort to prepare, adapt and test the training for each audience has been made and this program has been very successful. It involved clinicians also.

1.5. Georgia

In accordance with the Law of Georgia on Public Health, specialists in public health and epidemiology are educated at the higher education level, holding a bachelor's degree or higher, as determined by the Ministry of Health.[11] Over the last three decades, the Georgian health system underwent substantial transformations post-1991 independence, with reforms in medical education witnessing the establishment of private medical schools and medical faculties within public universities.[12] The subject of

"environmental health" or "hygiene" is integrated into the curricula of these faculties and specializations, predominantly during the initial three years of university education.

Tbilisi State Medical University is the exclusive institution granting a bachelor's degree in public health, with master's degree programs in public health offered by several institutions. However, Tbilisi State Medical University stands alone in providing master's degree programs specifically in epidemiology and Environmental Medicine.[13]

The National Center for Disease Control and Public Health (NCDC) plays a pivotal role in Georgia's public health, contributing significantly to health care system development. The NCDC engages in disease prevention, laboratory diagnostics, and responds to public health emergencies, managing state public health programs. It oversees health-related databases, including the R. Lugar Center for Public Health Research, a BSL-3 facility.

The MediPIET and EEFTP field epidemiology training programs operate under the NCDC. MediPIET, a two-year fellowship, combines practical training with mentoring to enhance competencies in intervention epidemiology. The SC-FELTP, since 2009, has trained professionals from Georgia and neighbouring countries, recognized as a cornerstone of health diplomacy.[14,15]

The NCDC's 2018-2022 Strategic Plan prioritizes coordinated, evidence-based policies in environmental health, emphasizing inter-sectoral cooperation. The plan advocates for the integration of information systems, development of environmental health indicators, and adherence to the "Ostrava Declaration" for sustainable development goals.[16] Financial constraints and limited access to continuing medical and nursing education pose significant challenges, as highlighted in the National Health Care Strategy of Georgia (2022-2030). [17] Continuous professional development remains an issue for human resources in the health sector, with a focus on modernizing theoretical and practical skills. The NCDC, supported by the US CDC, is actively developing a strategy for public health human resource development to address these challenges.

1.6. Italy

As an example of the value of inter-disciplinary collaboration for production of research that is valid and suitable for application to services for protection of public health, we refer to the topic of heat waves and pollen concentration in the atmosphere. The Environmental Protection Agency of Emilia Romagna Region made some of the first internationally recognized contributions on environmental health prevention in the fields of heat waves and pollen diffusion. This was achieved through the collaboration of physicists, mathematicians, agronomists, and physicians. The integration of these different disciplines and competences proved to be necessary and effective. Following the publication of these experiences, two forecasting systems for heatwaves and pollens were implemented in the Emilia Romagna Region and later transferred to a national setting. [18, 19]

1.7. Poland

In Central and Eastern European countries public health activities are mainly focused on the prevention of communicable diseases but in recent times to more and more extent also non-communicable ones. The orientation on environmental problems is usually present when clear problems arrived e.g. smog, floods, or extremely high temperature.

In Poland, both medical personnel (physicians and nurses), as well as nonmedical public health officials, receive a strong background during pre-diploma training in preventive medicine. Environmental causes of

common diseases, as well as cultural, social, and economic health determinants, are well covered. Health promotion in physicians, nurses, and midwives' curricula is one of the major skills to develop. On other hand, EH is rather to a smaller extent represented in the curriculum of physicians. However, in case nurses and midwives - the emphasis is placed on the wide spectrum of factors related to the patient's home, education, and work.

During post-graduate training of non-medical public health professionals, the EH knowledge was covered in the curriculum only if they continue their carrier development in selected post-graduation specialties like "Environmental health", "Public health" or "Epidemiology". In the case of physicians and nurses, the topics of EH are linked mainly to epidemiology training.

In Poland, the Ministry of Health is the main institution to address all governmental decision in the area of EH. The Chief environmental health consultant and the regional ones are the first lines of advisers.

The focus is mainly on air pollution, but growing attention is given to climate change issues. In relation to first one, Ministry is advised also by Permanent Commission on Health Effects of Air Pollution. Specific issues related to other issues like e.g. electromagnetic fields, indoor air, or climate change are referred to the National Institute of Public Health (NIZPH_PZH), the Nofer Institute of Occupational Medicine (NIOM), the Chemical Bureau or the Institute of Environmental Protection. The monitoring of drinking and bathing water quality is run by District Sanitary Inspectorate and supervised by Chief Sanitary Inspectorate (GIS).

1.8. Turkey

In Turkey, various initiatives have been undertaken to integrate planetary health lessons into educational curricula. Higher education institutions offer courses such as "Environmental Education," "Turkey's Environmental Problems," "Environmental Philosophy," and "Environmental Law." The Council of Higher Education (YÖK) has updated teacher training programs, introducing a new course, "Early Childhood Environmental Education," in the Preschool Education undergraduate program. [20]

Six different institutes in Turkey have published 78 doctoral theses related to education, covering areas like environmental ethics, engineering, law, problems, ecology, and natural resource management. For example, many of these areas are required for professionals to effectively address illegal waste dumping practices.[21] Environmental education aims to bring about attitude changes and behaviors by providing both positive and negative information about the environment. It intersects with various fields, contributing to solutions for existing environmental problems. [22]

Environmental education themes in programs encompass conscious consumerism, improving environmental awareness, recognizing environmental problems, disaster preparedness, environmental design, understanding societal and industrial impacts on the environment, and preserving cultural assets. Health Services Vocational High Schools, particularly the Environmental Health (EH) program, train technicians to address environmental conditions affecting human health. [23,24]

Environmental medicine is an emerging clinical specialty, evolving within branches like paediatrics, geriatrics, physiology, biochemistry, and epidemiology. Family Physicians/General Practitioners (FPs/GPs) consider planetary health as part of their holistic approach. Studies evaluating the effectiveness of Environmental and Planetary Health (EPH) education in Turkey have been conducted, with future efforts exploring student-centered teaching methods to enhance environmental sensitivity and attitudes. [25, 26, 27, 28, 29, 30]

1.9. The UK

Core elements of what constitutes good public health practice have a strong focus in the Faculty of Public Health curriculum, so that public health trainees will have an opportunity to demonstrate in actual service practice both the confidence and competence necessary to go on to develop increasing levels of expertise in their subsequent, more specialised professional practice. Public health trainees are expected not only to know about good public health practice and show they can do it or apply it in a protected setting, but, over the length of the training programme, to undertake and do their daily work with required levels of knowledge and understanding and at increasing levels of complexity.

Areas of public health practice

The Faculty of Public Health of the Royal College of Physicians defines and supports training to achieve broad competencies in ten key areas of public health practice: [31]

- Use of public health intelligence to survey and assess a population's health and wellbeing;
- Assessing the evidence of effectiveness of interventions, programmes and services intended to improve health or wellbeing of individuals or populations;
    - Policy and strategy and evidence development, translation and implementation;
    - Strategic leadership and collaborative working for health;
    - Health promotion, determinants of health and health communication;
    - Health protection;
    - Health and care public health;
    - Academic public health;
    - Professional personal and ethical development;
    - Integration and application of competences for consultant practice..

A detailed description of the areas and the organisation has been carried by some Universities and Institutional embodies [32] and provides:

- the functional areas in which individuals, teams and organisations operate, to deliver on public health outcomes:
- statements that describes what functions and individual carry out in the course of the work. The combination of functions will vary from individual to individual, and from role to role;
- a benchmark or single point of reference for the UK workforce and their employer to help individuals to plan their own personal development, and to help employers to plan and develop their workforce;
- a tool to facilitate the generation of job description for new roles: templates for standard roles; and profiles for individual roles;
- a common reference for the review and development of standards of practice and curricula for training and education qualifications across all levels of the qualification's framework;
- a description of the public health functions and subfunctions in a way that could be presented through an accessible and easy to navigate interactive digital platform

Three-Dimensional Model of Learning

This model shows schematically the interrelationship between the knowledge base (shown on the side face in six domains), the learning outcomes (shown on the front face in the nine key areas of public health practice) and professional behaviours/public health contexts and learning phases (shown on the top face).

The model explores how each part of the three elements cross-cuts the other two. It aims at linking the knowledge base with learning outcomes within a public health context. [33]

Figure 1 Three-dimensional model Learning

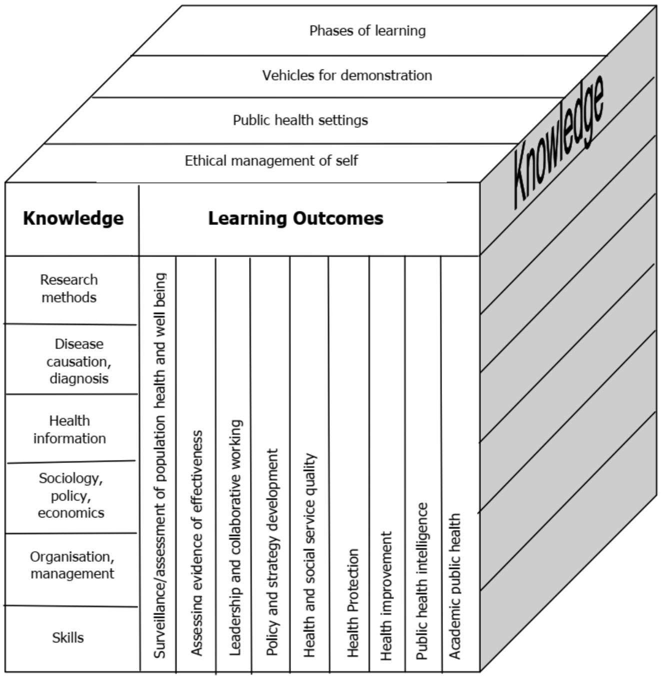

Climate change and sustainability appear as a cross-cutting theme in the UK 2022 curriculum, but the role of pollution and loss of biodiversity are not mentioned. Nevertheless, the overall approach has inspired the development of a set of training approaches in relation to environmental public health, which has produced training programmes, including a short course,[34] for staff who wished to develop their capacity to handle environmental epidemiology, toxicology and natural sciences for the purpose of health protection and improvement.[35]

1.10. United States of America

The US-CDC funds 33 state and local tracking programs as a part of the National Environmental Public Health Tracking Program (EPHT). Tracking Program successes stem from its network of experts and partners who are committed to improving health outcomes in relation to prevention of environmental hazards across the United States. Tracking Program grantees provide expertise, data, and tools to inform decisions about allocating resources, planning interventions, and evaluating efforts to protect and improve the health of their communities. Examples include the contribution of New York tracking programs to aid disaster response during hurricane Sandy, the Florida mercury biomonitoring project that informs campaigns for safe consumption of fish, the Maine tracking activity to map Lyme disease and tick distribution. [36]

The Children's Environmental Health Committee of the Environment Section of the American Public Health Association established a workgroup to develop a Children's Environmental Health curriculum. This group identified twelve critical competencies in children's environmental health (Table 1). [37] These competencies are addressed to public health students and professionals, to support their building of capacity in this area.

Table 1. Critical Competencies in Children's Environmental Health

Competency #	Competency
1	Assess a children's environmental health concern, risk, or potential exposure in a community and develop a briefing paper.
2	Present information to stakeholders about children's environmental health threats and prevention methods.
3	Develop, implement, and evaluate a community-based intervention to mitigate a children's environmental health threat.
4	Increase children's exposure to healthy natural environments.
5	Monitor and report child health indicators to the state or local public health department.
6	Communicate to the media promoting children's environmental health through traditional and nontraditional outlets (e.g., social media).
7	Identify how climate change and environmental exposures (e.g., pesticides) affect children's health (short and long term).
8	Be able to recognize or assess structural and systemic harms (e.g., built environment, climate change, risks associated with exposure) on children's health.
9	Identify federal, state, and local regulations as they relate to children's health and the environment.
10	Prepare and present testimony about children's health and the environment before local and state legislators.
11	Identify actions and evaluate yearly progress toward the reduction of greenhouse gas emissions and the carbon footprint of an organization (i.e., state or local health department).
12	Design environmental health guidelines that account for children's unique vulnerabilities and long-term susceptibility to health effects.

1.11. Zambia

Zambia's environmental public health framework relies on national policies and legislation, allocating responsibilities to specific government bodies. The National Policy on Environment (NPE) [38] serves as a comprehensive guide, aiming to ensure citizens' right to a health-supportive environment. The 2011 Environmental Management Act [39] established the Zambia Environmental Management Agency (ZEMA), tasked with protecting the environment and controlling pollution for the well-being of people, animals, plants, and ecosystems.

The Ministry of Health Zambia and Zambia National Public Health Institute (ZNPHI) oversee human health, while animal health is under the Ministry of Fisheries and Livestock, and plant health is primarily managed by the Ministry of Agriculture. Despite a robust policy framework, practical implementation of environmental public health measures is limited in Zambia, with exceptions such as water source monitoring by Ministry of Health environmental health technicians.

Zambia provides environmental health education at various levels, including the Africa Centre of Excellence for Infectious Diseases of Humans and Animals (ACEIDHA) at the University of Zambia, offering relevant post-graduate courses. Additionally, the Zambia Field Epidemiology Training Programme (ZFETP), in collaboration with global health agencies, introduced a five-day training module on environmental epidemiology in 2022. This module aims to familiarize trainees with environmental public health practices, covering risk identification, exposure assessment, and investigation methodologies for non-communicable diseases associated with environmental hazards. [40, 41, 42, 43]

2. Supranational experiences and lessons learnt

2.1. International Network on Public Health and Environment Tracking – INPHET

INPHET was formed to address common interests and activities in numerous countries to advance EPHT and to enhance use of these data to inform public health actions.[44] INPHET is a community of practice initially developed in 2011-2013 and formally established in 2014, which aims to increase the quality, availability, comparability, integration, and use of environmental monitoring and exposure and health surveillance data, enhance national tracking capacity by providing a mechanism for members to share experiences, seek input on challenges, and provide opportunities for mentorship, increase cross-national collaboration on common issues, advance systematic analyses of environmental health data such as monitoring trends and burden of environmentally-related diseases, monitoring environmental precursors of disease and health

Integrating, analysing and interpreting geospatial and temporal trends and relationships between environmental hazards, exposure and health data, identifying populations at risk from environmental hazards, implementing and evaluating intervention and prevention strategies, and informing public health decision-makers. INPHET members include Health departments in Ministries of health, International and national public health organizations (e.g., public health practitioners, civil servants), Non-governmental organizations, environmental agencies, and related organizations, Data stewards, Academic Institutions (e.g., researchers, scientists, educators). INPHET has organised several events and shared resources and initiatives on topics relevant to its aims globally.

2.2. Lessons across Europe

That environmental health aspects require a broad perspective encompassing pollution alongside ecology and climate change was highlighted in a book for policy makers in the European region of WHO for effective operation of national public health systems, though several processes are already available to make progress, including training. [45] An EU-funded project named "A European network for the training and development of public health (environment) physicians (PHEEDUNET) was carried out from 2008 to 2011 with the following aims: to identify training and continuing professional development (CPD) needs of Public Health (Environment) (PH(E)) physicians in all EU countries and assess levels of variation in access to training courses between countries; to consider how courses can be coordinated to improve access to training and CPD in different countries; and to develop a proposal for joint training and CPD programme courses across EU countries that the network should be coordinated. The main findings were summarised in documents on (i) definitions distinguishing environmental public health from environmental medicine and environmental health, [4] (ii) curricula available for registration as specialist in public health with subspecialty “Environment”,[5] and (iii) procedures available for registration as subspecialist in this field. [6] Although individual countries have since further developed their practice and procedures, PHEEDUNET remains relevant as a review of procedures leading to recognition of a professional, legally recognised role

in society as environmental public health specialist, comparing progress in most countries across the region.

2.3. Lessons from World Health Organisation WHO

Online resources

The WHO's Department of Environment, Climate Change and Health has provided several online training resources developed by WHO and UN partners on a broad range of topics: air pollution and energy; climate change; chemical safety; children's environmental health; radiation; healthy and safe workplaces; water, sanitation and hygiene.[46] The audience comprises policy makers as well as health professionals. The materials include brief e-learning lasting 2 hours [47] , webinar series comprising 7 sessions, [48] virtual campuses assigned 30 hours,[49] comprehensive web-based academies.

WHO Academy for Practitioners in climate science

The WHO Academy for Practitioners in climate science offers a curriculum titled Basic Concepts on Climate and Climate Services for Health. [50] Designed for environmental public health (EPH) practitioners with an epidemiology background, it imparts fundamental knowledge in climate science, forecasting, data analysis, and understanding climate-related health impacts. The curriculum introduces climate services, covers modeling approaches, and addresses spatial and temporal patterns of health risks. It also focuses on implementing climate-informed decision support tools, anticipating the escalating threats from climate change.

Additionally, WHO is developing air pollution and health training material for the health workforce. Piloted in Kumasi, Ghana, this training includes modules on ambient and household air pollution, health effects of air pollution, and the role of the health workforce. The workshop, held in collaboration with Ghana Health Service and WHO offices, utilized a train-the-trainer approach, engaging participants in interactive sessions and field visits to air pollution hotspots.

Air pollution and health training for the health workforce – piloting experience in Kumasi, Ghana

WHO is developing comprehensive air pollution and health training to empower the health workforce in addressing air pollution's health effects and mitigating risks through effective communication. The material comprises training modules, a trainer manual, and clinical case scenarios. The pilot in Kumasi, Ghana, marked WHO's first-ever air pollution and health training for the health workforce, gathering nearly 50 participants, including physicians and public health officers. Held from June 6 to 10, 2022, in collaboration with Ghana Health Service and WHO offices, the workshop employed a train-the-trainer approach. Participants gained knowledge on air pollution and its health impact, becoming advocates and trainers for other health professionals. The workshop included four modules covering ambient and household air pollution, health effects overview, and the health workforce's role. Interactive sessions, activities, and field visits to Kumasi's air pollution hotspots enriched the learning experience.

2.4. Environmental health in International Health Regulation implementation

The International Health Regulations 2005 (IHR) provide a unique public health framework that enable countries to better prevent, prepare for and respond to public health events and emergencies of potential international concern. [51] IHR 2005 is not limited to any specific disease or manner of transmission, but covers all diseases and events of international public health concern, including those linked to chemical and other environmental hazards.

Several technical and professional figures are relevant to achievement of such goals, including Field Epidemiology Training Programs (FETPs) and laboratory capacity. The IHR requires countries to establish core capacities for surveillance and response to chemical and other environmental events and exposures at all administrative levels in a country. A systematic review on lessons learnt from implementation of IHR recognised the need for response and surveillance plans to be reviewed and updated regularly to respond to all hazards: zoonotic, food safety, chemical, radionuclear and antimicrobial resistance threats.[52] To address this, an environmental epidemiology training module has been developed for FETP in a few countries, including Ghana, Pakistan, UK, Zambia, with a broad scope including strengthening public health capacity in relation to chemicals, environmental precursors of infection, and climate change.[53]

References

1. National Health and Climate Strategy. (2023) Department of Health and Aged Care, Australian Government. Available at: https://www.health.gov.au/our-work/national-health-and-climate-strategy
2. Vardoulakis, S., Johnston, F. H., Goodman, N., Morgan, G. G., & Robinson, D. L. (2024). Wood heater smoke and mortality in the Australian Capital Territory: a rapid health impact assessment. Medical Journal of Australia, 220(1), 29-34.
3. Matthews, V., Vine, K., Atkinson, A. R., Longman, J., Lee, G. W., Vardoulakis, S., & Mohamed, J. (2023). Justice, culture, and relationships: Australian Indigenous prescription for planetary health. *Science*, *381*(6658), 636-641
4. Kreis IA, Leonardi GS, Murray VSG, Ortega Garcia JA, van Loenhout J, Ohnsorge P. PHEEDUNET Document 1 (16 pages). Definitions. A European network for the training and development of public health (environment) physicians. EU Public Health Executive Agency Project No: 2006335. 30 Mar. 2009. Available at: https://www.researchgate.net/publication/336217074_PHEEDUNET_Document_1_Definitions_16_pages_A_EUROPEAN_NETWORK_FOR_THE_TRAINING_AND_DEVELOPMENT_OF_PUBLIC_HEALTH_ENVIRONMENT_PHYSICIANS
5. Kreis IA, Murray V, Leonardi G, van den Hazel P. PHEEDUNET Document 3: Curricula for Registration. A European network for the training and development of public health (environment) physicians. EU Public Health Executive Agency Project No: 2006335. 31 March 2010. Available at: https://www.researchgate.net/publication/336217222_PHEEDUNET_Document_3_609_pages_Curricula_A_European_Network_for_the_Training_and_development_of_Public_Health_Environment_Physicians. Accessed 23/10/2023
6. Kreis IA, Murray V, Leonardi G, van den Hazel P. 2010. PHEEDUNET Document 4: Registration (procedures for registration as specialist in environmental public health). A European network for the training and development of public health (environment) physicians. EU Public Health Executive Agency Project No: 2006335. 31 March 2010. DOI: 10.13140/RG.2.2.11366.98886
7. Peter Van den Hazel, Ilse Loots, Greet Schoeters, Jean Pauluis, and Marie-Christine Dewolf, 2014. Training and specialisation for health care professionals in environmental and health medicine (call for offer – ref. n° DVZ/cel L&G/YN/2011-001) 132pp).
8. Webb, J., S. Raez-Villanueva, P. D. Carriere, A. A. Beauchamp, I. Bell, A. Day, S. Elton, M. Feagan, J. Giacinti, J. P. Kabemba Lukusa, C. Kingsbury, P. A. Torres-Slimming, M. Bunch, K. Clow, M. K. Gislason, M. W. Parkes, E. Jane Parmley, B. Poland, and C. Vaillancourt. 2023. 'Transformative learning for a sustainable and healthy future through ecosystem approaches to health: insights from 15 years of co-designed ecohealth teaching and learning experiences', Lancet Planet Health, 7: e86-e96
[9] Redvers, J. (2020). "The land is a healer": perspectives on land-based healing from Indigenous practitioners in northern Canada. International Journal of Indigenous Health, 15(1), 90-107.
10. Universite Paris-Est Creteil – Airparif – Aireducation accessed on 22/7/2024 at https://www.airducation.eu/
11. Georgian Ministry of Health (Gerogian) accessed on 22//2024 at https://matsne.gov.ge/document/view/21784
12. Ruadze E, Cherkezishvili E, Roma E, et al. Multistakeholder perspectives on the strengthening and embedding of mandatory continuing medical education in Georgia: a qualitative study. BMJ Open 2021;11:e052686. doi:10.1136/bmjopen-2021-052686
13. National Centre for Educational Quality Enhancemente (Georgian) accessed 22/7/2024 at https://naec.ge/uploads/postData/20-22/gzamkvlevi%20-2022.pdf
14. MediPied accessed on 22/7/2024 at https://medipiet.isciii.es/portfolio/layout/
15. Tephinet accesed on 22/72024 T https://www.tephinet.org/training-programs/south-caucasus-field-epidemiology-and-laboratory-training-program
16. NCDC accesd on 22/7/2024 at https://www.ncdc.ge/#/pages/file/fade4aa1-397c-4b59-8bc4-dd097ff81228
17. Georgia National Health Strategy 2022 – 2030 https://matsne.gov.ge/document/view/5453716?publication=0
18 .Zauli Sajani, S., Tibaldi, S., Scotto, F., & Lauriola, P. (2008). Bioclimatic characterisation of an urban area: a case study in Bologna (Italy). International journal of biometeorology, 52, 779-785.
19. Zauli Sajani, S., Tibaldi, S., Scotto, F., & Lauriola, P. (2008). Bioclimatic characterisation of an urban area: a case study in Bologna (Italy). International journal of biometeorology, 52, 779-785.
20. Study in Turkiye accessed on 22/7/20224 https://www.studyinturkey.gov.tr/StudyinTurkey/ShowDetail?rID=Ec/rgHEN8Zg=&&cId=PE4Nr0mMoY4=
[21] Gokdemir, O., Randenikumara, S., Floss, M., Rochfort, A., & Astier Peña, M. P. (2023). Illegal waste dumping practices: Where does all the garbage go?. Environmental Forensics, 1-4.
22.SÖNMEZ D, HASTÜRK HG, ÜNAL BB. TÜRKİYE'DEğitim VeÖğretim Alaninda ÇevEğitimiİİlgili DoktorTezleriniİncelenmesi. J Hist Sch. 2022;LVI(LVI):298–321.

23. Kahyaoğlu M. Review TÜRKİYE ’ DE ÇEVRE EĞİTİMİ ÜZERİNE YAPILAN ARAŞTIRMALAR : BİR İÇERİK ANALİZİ ÇALIŞMASI A Study on Environmental Education Research in Turkey : A Content Analysis Study. Marmara Coğrafya Derg / Marmara Geogr. 2016;(July):50–60.
24. Özcan S, Ek HN, Kılınç N. Opinion And Attitudes Towards Environmental Problems of Environmental Health Pre-License Program’s Students in Turkey. Kastamonu Eğitim Derg. 2018;26(3):1–10.
25 WHO- Air pollution and health: an introduction for health workers accessible at https://openwho.org/courses/air-pollution-health-workers
26. Bakırcı H, Artun H. A CASE STUDY COMPARING ENVIRONMENTAL EDUCATIONAL POLICIES OF DIFFERENT COUNTRIES. Elektron Sos Bilim Derg Electron J Soc Sci. 2011;37:202–23
27. Cubaka VK, Dyck C, Dawe R, Alghalyini B, Whalen-Browne M, Cejas G, et al. A global picture of family medicine: The view from a WONCA Storybooth. BMC Fam Pract. 2019;20(1):1–9.
28. Lauriola P, Serafini A, Santamaria M,et al. Family doctors to connect global concerns due to climate change with local actions: State-of-the art and some proposals. World Med Heal Policy. 2021;13(2):199–223.
29. Özcan S, Ek HN, Kılınç N. Opinion And Attitudes Towards Environmental Problems of Environmental Health Pre-License Program’s Students in Turkey. Kastamonu Eğitim Derg. 2018;26(3):1–10.
30. Aykaç Koçak A, Yıldırım B. Evaluation of articles related to environmental health published in nursing and health sciences faculty journals between 2010 and 2019 In Turkey. Med Sci. 2021;16(1):1–8.
31. UK Faculty of Public Health. Public Health Specialty Training Curriculum 2022. Available at: https://www.fph.org.uk/media/3537/public-health-training-curriculum-2022-final.pdf
32. Public Health Skills and Knowledge Framework 2016 (publishing.service.gov.uk) https://assets.publishing.service.gov.uk/government/uploads/system/uploads/attachment_data/file/584408/public_health_skills_and_knowledge_framework.pdf
33. UK Faculty of Public Health. Functions and standards of a public health system. 2022. Available at: https://www.fph.org.uk/media/3031/fph_systems_and_function-final-v2.pdf
34. Kreis I, Leonardi G, Murray V. 2010 "Lessons from six years of the HPA/LSHTM course: Introduction to Environmental Epidemiology. . ." In Chemical Hazards and Poisons Report, page 51.
35. Spiby, J. 2006. "Developing Competencies in Environmental Public Health." In Chemical Hazards and Poisons Report. https://assets.publishing.service.gov.uk/media/5a7c9b2aed915d12ab4bbea4/rep_Chapr6Feb2006.pdf
[36] US-CDC. (2024) Tracking Success Stories. Available at: https://www.cdc.gov/nceh/tracking/successstories.htm
[37] Del Rio, M., Lasley, P., Tallon, L., Kauth,J-M., Bare, G., & Etzel, R. A. (2023). Critical Competencies in Children's Environmental Health. Journal of Environmental Health, 85(6), 26-29.
38. Zambia National Policy on Environment, 2009 - http://zm.chm-cbd.net/implementation/legislation/policies-related-environment-and-biological/national-policy-environment-npe/npe_main_body_2009.doc
39. Zambia Environmental Management Act 2011 - https://www.parliament.gov.zm/sites/default/files/documents/acts/Environmetal Mangement Act 12 of 2011.pdf
Yabe J, Nakayama SM, Nakata H, Toyomaki H et al. Current trends of blood lead levels, distribution patterns and exposure variations among household members in Kabwe, Zambia. Chemosphere. 2020 Mar;243:125412. doi: 10.1016/j.chemosphere.2019.125412. Epub 2019 Nov 19. PMID: 31995873.
40. Yabe J, Nakayama SM, Nakata H, Toyomaki H et al. Current trends of blood lead levels, distribution patterns and exposure variations among household members in Kabwe, Zambia. Chemosphere. 2020 Mar;243:125412. doi: 10.1016/j.chemosphere.2019.125412. Epub 2019 Nov 19. PMID: 31995873.
41. Yabe J, Nakayama SM, Nakata H, Toyomaki H et al. Current trends of blood lead levels, distribution patterns and exposure variations among household members in Kabwe, Zambia. Chemosphere. 2020 Mar;243:125412. doi: 10.1016/j.chemosphere.2019.125412. Epub 2019 Nov 19. PMID: 31995873.
42. Bose-O'Reilly S, Yabe J, Makumba J, Schutzmeier P, Ericson B, Caravanos J. Lead intoxicated children in Kabwe, Environ Res. 2018 Aug:165:420-424. doi: 10.1016/j.envres.2017.10.024. Epub 2017 Oct 28
43. AFRICA CENTRE OF EXCELLENCE FOR INFECTIOUS DISEASES OF HUMANS AND ANIMALS (ACEIDHA) https://aceidha.unza.zm/
44. INPHET, International Network on Public Health and Environment Tracking. Available at www.inphet.org.
45. Leonardi, G., and B. Rechel. 2014. 'Environmental Health.' in, Rechel B, McKee M (Eds). Facets of Public Health in Europe. ISBN: 9780335264209 (Open University Press. European Observatory on Health Systems and Policies Series).
46. WHO. 2023. 'Environmental health training', World Health Organization. Department of Environment, Climate Change and Health. https://www.who.int/teams/environment-climate- change-and-health/training/.
47. WHO. 2023. 'Environment, climate change and health for practitioners and actors guiding policy change', World Health Organisation. OpenWHO. https://openwho.org/courses/health-environment-climate-change.
48. WHO. 2021. 'Transitioning to Clean Cooking', World Health Organisation and Clean Cooking Alliance. https://www.who.int/initiatives/health-and-energy-platform-of-action/hepa-repository/webinar-series-transitioning-to-clean-cooking.

49. WHO. 2023. WHO Chemical Road Map on Chemical Products (English version). Available at: https://www.campusvirtualsp.org/en/course/who-chemical-road-map-chemical-products-english-version-2021
50. Elena Villalobos Prats, Tara Neville, Kari C Nadeau, Diarmid Campbell-Lendrum, WHO Academy education: globally oriented, multicultural approaches to climate change and health, The Lancet Planetary Health, Volume 7, Issue 1, 2023, Pages e10-e11, ISSN 2542-5196,
https://doi.org/10.1016/S2542-5196(22)00252-2.
51. WHO (2016) - International Health Regulations, 2005,
http://apps.who.int/iris/bitstream/10665/246107/1/9789241580496-eng.pdf?ua=1 , accessed 17/11/2023).
52. Suthar, A.B., Allen, L.G., Cifuentes, S., Dye, C. and Nagata, J.M., 2018. Lessons learnt from implementation of the International Health Regulations: a systematic review. Bulletin of the World Health Organization, 96(2), p.110. doi: 10.2471/BLT.16.189100).
53. Leonardi G, Kaburi BB, Peacocke E, Nyadedzor C, Kenu E, Afari EA, Pett J, Sinyange N,Brooke N, Izon-Cooper L, Hams R, Iqbal N, Thomas E, Crabbe H. 2022. "The role of field epidemiology in strengthening public health capacity for chemical and environmental hazards in Ghana and Zambia." In ISEE 2022: 34th Annual Conference of the International Society of Environmental Epidemiology. ISEE Conference Abstracts.
